# Tailored immunosuppression after kidney transplantation - a single center real-life experience

**DOI:** 10.1186/s12882-020-02137-5

**Published:** 2020-11-23

**Authors:** Miriam Good-Weber, Malgorzata Roos, Thomas F. Mueller, Barbara Rüsi, Thomas Fehr

**Affiliations:** 1 Division of Nephrology, University Hospital Zurich, Zurich, Switzerland; 2 Department of Biostatistics, Epidemiology, Biostatistics and Prevention Institute, University Zurich, Zurich, Switzerland; 3 HLA Typing Laboratory, University Hospital Zurich, Zurich, Switzerland; 4 Department of Internal Medicine, Cantonal Hospital Graubünden, Loestrasse 170, 7000 Chur, Switzerland

**Keywords:** Kidney transplantation, Induction therapy, Thymoglobulin, Donor-specific antibodies, ABO-incompatibility, Rejection

## Abstract

**Background:**

Kidney allograft survival continuously improved with introduction of novel immunosuppressants. However, also immunologically challenging transplants (blood group incompatibility and sensitized recipients) increase. Between 2006 and 2008, a new tailored immunosuppression scheme for kidney transplantation was implemented at the University Hospital in Zurich, together with an ABO-incompatible transplant program and systematic pre- and posttransplant anti-human leukocyte antigen (HLA) antibody screening by Luminex technology. This study retrospectively evaluated the results of this tailored immunosuppression approach with a particular focus on immunologically higher risk transplants.

**Methods:**

A total of 204 consecutive kidney transplantations were analyzed, of whom 14 were ABO-incompatible and 35 recipients were donor-specific anti-HLA antibodies (DSA) positive, but complement-dependent cytotoxicity crossmatch (CDC-XM) negative. We analyzed patient and graft survival, acute rejection rates and infectious complications in ABO-compatible versus -incompatible and in DSA positive versus negative patients and compared those with a historical control group.

**Results:**

Overall patient, death-censored allograft survival and non-death-censored allograft survival at 4 years were 92, 91 and 87%, respectively. We found that (1) there were no differences between ABO-compatible and -incompatible and between DSA positive and DSA negative patients concerning acute rejection rate and graft survival; (2) compared with the historical control group there was a significant decrease of acute rejection rates in sensitized patients who received an induction with thymoglobulin; (3) there was no increased rate of infection among the patients who received induction with thymoglobulin compared to no induction therapy.

**Conclusions:**

We observed excellent overall mid-term patient and graft survival rates with our tailored immunosuppression approach. Induction with thymoglobulin was efficient and safe in keeping rejection rates low in DSA positive patients with a negative CDC-XM.

**Supplementary Information:**

The online version contains supplementary material available at 10.1186/s12882-020-02137-5.

## Background

Allograft survival after kidney transplantation has substantially improved with modern immunosuppressive drugs - particularly in the first year post-transplant -, but still 5–10% of patients suffer from acute rejection, which overall leads to a reduced graft survival [1–3]. General risk factors for acute rejection and a shortened graft survival are prolonged cold ischemia time, body mass index (BMI) > 25 kg/m^2^, AB0 incompatibility, number of human leukocyte antigen (HLA) mismatches and retransplantation [4]. Furthermore, several studies demonstrated that the presence of donor-specific anti-HLA antibodies (DSA) increased the risk of antibody-mediated rejection (AMR) and was deleterious for allograft survival [5–9]. To avoid immunologically incompatible transplantation (AB0 incompatibility, DSA positive patients) paired kidney donation was introduced in many countries including Switzerland, a procedure allowing conversion of incompatible to compatible pairs [10]. If this is not possible risk-adapted immunosuppression and/or desensitization represent alternative options [11]. Depending on the immunological risk, this can be performed using anti-thymocyte globulin such as thymoglobulin or rituximab induction and/or intravenous immunoglobulin with or without plasmapheresis/ immunoadsorption [12]. Previous studies showed that induction with thymoglobulin leads to a reduction of acute rejections in DSA positive, complement-dependent cytotoxicity crossmatch (CDC-XM) negative patients [13–15].

Based on these observations a new tailored immunosuppression scheme was implemented at the University Hospital Zurich between 2006 to 2008 (Table 1). For DSA positive patients a risk-adapted immunosuppression protocol including thymoglobulin induction was introduced, alongside with the universal pre- and posttransplant monitoring of anti-HLA antibodies using the Luminex technology; furthermore, a program for ABO-incompatible transplantation using rituximab induction was started.

**Table 1:** Tailored immunosuppressive regimens in the Zurich renal transplant program before 2006 and from 2008 to 2011

Regimen	Patient group	Transplantation until 2006	Transplantation from 2008
1	first transplantation without anti-HLA immunization	cyclosporine, MMF, prednisone	cyclosporine, MMF, prednisone
2a	retransplantation and/or anti-HLA immunization, but without DSA	basiliximab, tacrolimus, MMF, prednisone	basiliximab, tacrolimus, MMF, prednisone
2b	recipient with DSA and negative CDC cross-match	basiliximab, tacrolimus, MMF, prednisone	thymoglobulin, tacrolimus, MMF, prednisone
3	donor risk (age above 75, cold ischemia time > 24 h, DCD)	thymoglobulin, tacrolimus delayed (day 5), MMF, prednisone	thymoglobulin, tacrolimus delayed (day 5), MMF, prednisone
4	ABO blood group incompatibility	(not performed)	rituximab + IADS, tacrolimus, MMF, prednisone

*CDC* Complement-dependent cytotoxicity, *DCD* Donor after circulatory death, *DSA* Donor-specific antibody, *IADS* Immunoadsorption with Glycosorb column, *MMF* Mycophenolate-mofetil

In this retrospective study we investigated in a single center real life setting how efficient this new tailored immunosuppression strategy was in terms of overall patient and graft survival after 4 years, in avoiding acute rejections in patients with preformed DSA compared to non-sensitized patients and in ABO-incompatible transplantations, and we compared the results in sensitized patients with DSA to the period prior to the introduction of this new immunosuppression scheme.

## Methods

### Patients

Between October 2008 and March 2011 a total of 219 kidney transplantations were performed at the University Hospital of Zurich. These patients were retrospectively included in our study. Pediatric patients were excluded, because they had their follow-up at the Zurich University Children’s Hospital (*n* = 10). Also excluded were patients, who had a combination of kidney and liver transplantation (*n* = 5), because of the postulated immunomodulatory effects of the liver graft [16]. Therefore, 204 patients (including combined kidney/pancreas and kidney/islet transplants) could be evaluated. Observation time was between 0 and 61.4 months (Median 41.2, 25% quartile: 33.6, 75% quartile: 48.6). This retrospective analysis was approved by the local ethics committee of the Canton of Zurich (KEK-10: 2012–0247).

### Screening for viral infections

BK polyomavirus screening by urinary polymerase chain reaction (PCR) was performed at 0, 3, 6, 9, 12, 18 and 24 months after transplantation. A cytomegalovirus (CMV) preemptive therapy approach was chosen at our center, with a clearly defined schedule for CMV PCR monitoring depending on the serostatus of donor and recipients [17]. No systematic screening for Epstein-Barr virus was performed.

### Immunosuppressive regimen

The revised standard immunosuppressive regimens starting from summer 2008 are summarized in Table 1.

For patients with DSA the induction therapy with basiliximab (standard dose of 2 × 20 mg) was replaced by anti-thymocyte globulin (thymoglobulin). Patients receiving an organ from a donor with risk factors for delayed graft function (higher age, longer cold ischemia time, donors after circulatory death) also received thymoglobulin in association with a delayed start of tacrolimus. The total dose of thymoglobulin was 1.5 mg/kg body weight on 5 consecutive days (i.e. the maximum dose was 7.5 mg/kg body weight). In case of profound lymphopenia the dose was reduced, but all patients received at least 3 doses. Outside of the regular immunosuppression schemes, three patients received de novo everolimus with sotrastaurin in the context of a clinical study protocol [18]. These three patients remained included in the overall analysis of patient and graft survival. For patients with ABO incompatibility the induction therapy was performed with rituximab. One single dose of rituximab (375 mg/m2) was given 4 weeks before transplantation. The initial steroid dose was identical for all protocols: solumedrol 500 mg i.v. on day 0, prednisone 100 mg p.o. on postoperative day 1 + 2, prednisone 0.5 mg/kg body weight p.o. from day 3 to day 14. A standardized tapering protocol was followed thereafter. Dosing of immunosuppressive drugs was standardized and tightly supervised. In combination with mycophenolate-mofetil (MMF) the target blood levels for cyclosporine were: 200–250 ng/ml (week 0–5), 180–220 ng/ml (week 6–11), 150–200 ng/ml (week 12 - month 5), 100–160 ng/ml (month 6–11), 80–120 ng/ml (month 12–17), 60–100 ng/ml (month 18–23) and 50–80 ng/ml thereafter. The doses for MMF in combination with cyclosporine were 1500 mg bid on day 0, 1500 mg bid on day 1–14 (dose reduction to 1000 mg bid in case of body weight < 50 kg or profound neutropenia) and 1000 mg bid from day 15. Also, in combination with MMF the target blood levels for tacrolimus were: 10–15 μg/l (week 0–5), 8–12 μg/l (week 6–11), 7–10 μg/l (week 12 - month 11), 6–8 μg/l (month 12–23), 4–6 μg/l thereafter. The doses for MMF in combination with tacrolimus were 1000 mg bid on day 0, 1000 mg bid on day 1–14 (dose reduction to 750 mg bid in case of body weight < 50 kg or profound neutropenia) and 750 mg bid from day 15.

### Anti-HLA antibody screening

Patients were screened for anti-HLA antibodies with Luminex LABScreen Mixed (One Lambda Inc., Canoga Park, CA, USA). This kit contains a panel of fluorescence-labeled microbeads coated with purified HLA antigens to identify anti-HLA class I or II IgG [19]. Test interpretation was performed using HLA Visual software (OneLambda Inc.) on the LABScan 100 flowcytometer (Luminex Inc., Austin, TX, USA).

### Single-antigen bead assay (SAB)

To identify the specificity of anti-HLA IgG antibodies, we performed the high-definition LABScreen Single Antigen (OneLambda) class I assay in LABScreen Mixed class I positive individuals and a class II assay in LABScreen Mixed class II positive individuals [20]. For result interpretation, Labscan 100 software (One Lambda) was used. The cut-off for a positive result was set at 500 mean fluorescence intensity (MFI) according to the manufacturer’s instruction.

A patient was classified as DSA positive when at least one DSA with MFI > 500 was detected. DSA were accepted up to MFI 10`000, when CDC-XM was negative. No flowcytometry-XM was performed.

### Outcome parameters

This retrospective analysis focused on the efficacy of the new tailored immunosuppressive scheme in a single center analysis in terms of overall patient and allograft survival, in prevention of acute rejection and allograft loss in DSA positive patients in comparison to DSA negative patients and in ABO-incompatible transplantations. The results in DSA positive patients were compared to the period previous to the introduction of thymoglobulin induction. All clinically suspected rejections were confirmed by a renal allograft biopsy. Biopsy specimens were evaluated by light microscopy and immunofluorescence including C4d staining. The histologic classification followed the Banff 2007 criteria [21].

Secondary outcome parameters included graft function and the incidence of infectious complications as the main safety parameter. Graft function is indicated as chronic kidney disease (CKD) stage since calculation of estimated glomerular filtration rate (GFR) was changed from MDRD to CKD-EPI formula during the study period and therefore not comparable among all patients.

### Statistical methods

Data were registered in MS Excel and analyzed with SPSS version 22. Mean and standard deviations were calculated for continuous variables and relative frequencies for discrete variables. Differences in medians of a continuous variable between two groups were checked with the non-parametric Mann-Whitney test. Associations between two discrete variables were evaluated with the Chi2-test.

Survival analysis with Kaplan-Meier survival curves estimation was considered for patient survival, graft survival and rejection-free survival. Observations, where death of a patient occurred, were considered as uncensored, whereas surviving patients were censored at the last day of follow-up. A similar type of censoring definition was applied to graft survival and rejection-free survival. In addition, the log-rank and Breslow-Gehan tests were performed for discrete predictors. The impact of a continuous predictor on survival was estimated by the Cox-regression.

Results of the statistical analysis with *P*-value < 0.05 were referred as statistically significant.

## Results

### Patient population

Baseline characteristics of patients are reported in Table 2.

**Table 2:** General patient characteristics

Parameter	All patients (*n* = 204)	No DSA^a^ (*n* = 169)	With DSA^b^ (*n* = 35)	*P*-value
Recipient age (year), mean (SD)	51 (±13)	51 (±12)	49 (±14)	0.428
Female sex, n (%)	83 (40.7)	63 (37.3)	20 (57.1)	0.024
Primary renal disease, n (%)				0.517
Vascular/hypertensive	18 (8.8)	15 (8.9)	3 (8.6)	
Diabetic	36 (17.6)	33 (19.5)	3 (8.6)	
Glomerulonephritis/vasculitis	61 (29.9)	45 (26.6)	16 (45.7)	
Cystic	31 (15.2)	26 (15.4)	5 (14.3)	
Urological	17 (8.3)	14 (8.3)	3 (8.6)	
Tubulointerstitiell	9 (4.4)	7 (4.1)	2 (5.7)	
HUS/TTP	1 (0.5)	1 (0.6)	0 (0.0)	
Others	8 (3.9)	7 (4.1)	1 (2.9)	
Indefinite	23 (11.3)	21 (12.4)	2 (5.7)	
Dialysis, n (%)	173 (84.8)	141 (83.4)	32 (91.4)	0.175
Number of transplantation, n (%)				< 0.001
First	172 (84.3)	154 (91.1)	18 (51.4)	
Second	30 (14.7)	15 (8.9)	15 (42.9)	
Third	2 (1.0)	0 (0.0)	2 (5.7)	
Donor type, n (%)				0.547
Living	69 (33.8)	57 (33.7)	12 (34.3)	
Deceased	135 (66.2)	112 (66.3)	23 (65.7)	
Type of transplantation, n (%)				0.195
Kidney	180 (88.2)	146 (86.4)	34 (97.1)	
Kidney + pancreas	21 (10.3)	20 (11.8)	1 (2.9)	
Kidney + pancreatic islets	3 (1.5)	3 (1.8)	0 (0.0)	
Blood group incompatibility, n (%)	14 (6.9)	11 (6.5)	3 (8.6)	0.443
HLA mismatches, mean (SD)	4.6 (±1.9)	4.5 (±1.9)	5.2 (±1.8)	0.228
Induction treatment, n (%)				
Thymoglobulin	47 (23.0)	22 (13.0)	25 (71.4)	< 0.001
Basiliximab	46 (22.5)	41 (24.3)	5 (14.3)	0.143
Rituximab (overall)	11 (5.4)	7 (4.1)	4 (11.4)	0.098
Rituximab alone	7 (3.4)			
Rituximab and basiliximab	3 (1.5)			
Rituximab and thymoglobulin	1 (0.5)			
Maintenance immunosuppression, n (%)				
Cyclosporine A	96 (47.1)	94 (55.6)	2 (5.7)	< 0.001
Tacrolimus	103 (50.5)	70 (41.4)	33 (94.3)	< 0.001

^a^*Abbreviations*: *DSA* Donor-specific antibody, *SD* Standard deviation, *n* Number, *HUS* Hemolytic uremic syndrome, *TTP* Thrombotic thrombocytopenic purpura, *HLA* Human leukocyte antigen

^b^Sensitizing events in the DSA positive group: previous transplantation 51.4%, pregnancy history 11.4%, previous blood transfusion 51.4%

The most frequent primary diseases leading to renal failure were glomerulonephritis or vasculitis (29.9%), diabetic nephropathy (17.6%) and cystic kidney disease (15.2%). Interestingly, in the group of DSA positive patients glomerulonephritis was almost twice as frequent compared to the DSA negative group.

With regard to induction therapy, the following agents were used: 47 patients received thymoglobulin (23.0%), 46 received basiliximab (22.5%), 7 received rituximab (3.4%) and 4 patients received a combination (2.0%, shown in Table 2). Overall, 14 patients underwent ABO blood group incompatible transplantation.

When looking closer at DSA, 35 (17.2%) among the 204 kidney recipients were DSA positive: 10 of them were only positive for class I, 18 were only positive for class II and 7 were positive for class I and II. Detailed information on specificity and titers of anti-HLA antibodies are given in Supplementary Table 1. As expected, the percentage of patients receiving a second or third transplant was significantly higher (48.6%) in the DSA positive group compared to the DSA negative group (8.9%). The majority of DSA positive patients received induction with thymoglobulin (74.3%), 7 received basiliximab (20%) and 4 rituximab (11.4%), in whom 3 also had a blood group incompatibility. Details on blood group incompatibilities and pre-transplant isoagglutinine titers are given in Supplementary Table 2.

### Patient survival

**Fig. 1:**
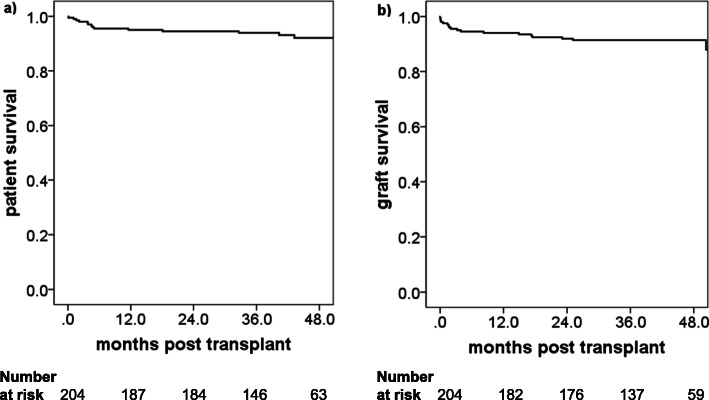
Outcome of kidney transplantation overall (*n* = 204 patients). Kaplan-Meier survival curves are shown for (**a**) patient survival and (**b**) death-censored graft survival

**Fig. 2:**
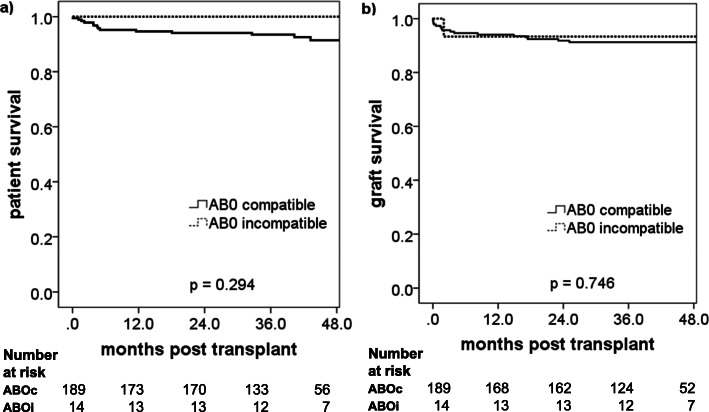
Outcome of kidney transplantation in ABO compatible (*n* = 189) versus ABO incompatible (*n* = 14) patients. Kaplan-Meier survival curves are shown for (**a**) patient survival and (**b**) death-censored graft survival

**Fig. 3:**
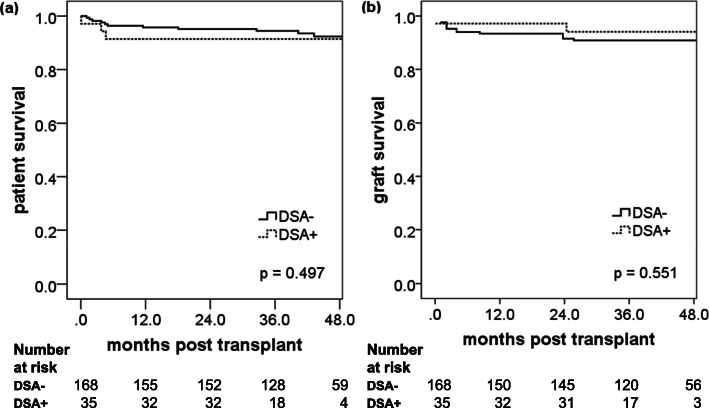
Outcome of kidney transplantation in DSA negative (DSA-, *n* = 168) and DSA positive (DSA+, *n* = 35) patients. Kaplan-Meier survival curves are shown for (**a**) patients survival and (**b**) death-censored graft survival

The overall survival of the 204 kidney transplants recipients was 95% at 1 year and 92% at 4 years (Fig. 1a). This result was similar to the survival rates in the Collaborative Transplant Study (CTS; transplant period 2000–2017; graph E-11012-0219). Among patients with blood group incompatibility all survived during the 4-year follow-up. Thereby, it needs to be considered, that all ABO incompatible transplantations were living donor transplants, which in general have a better outcome (Fig. 2a, *p* = 0.294). Also, between patients without and with DSA no difference in patient survival was observed, despite heavier immunosuppression in the latter (Fig. 3a, *p* = 0.497).

### Death-censored graft survival

The overall death-censored graft survival was 94% at 1 year and 91% at 4 years (Fig. 1b). These survival rates favorably compared to the respective rates in the CTS (transplant period 2000–2016; 1 year: 92%; 4 years: 83%; graph E-11011-0818). No difference was found between ABO incompatible and ABO compatible transplantations (Fig. 2b, *p* = 0.746) as well as between DSA positive and DSA negative recipients (Fig. 3b, *p* = 0.551). Especially the latter was a reassuring finding and confirmed the protective effect of thymoglobulin in sensitized patients with negative CDC cross-match – as suggested in the literature. This was further confirmed by an analysis of death-censored graft survival in the subgroup of thymoglobulin-treated patients only, which also showed no difference between DSA positive and DSA negative patients (data not shown).

### Rejection-free survival

The overall rejection-free survival was 67.4% at 1 year, again with no difference between DSA positive and DSA negative patients, as was the case also for ABO incompatible transplantations. Overall 17 patients (8.4%) experienced an AMR episode.

**Fig. 4:**
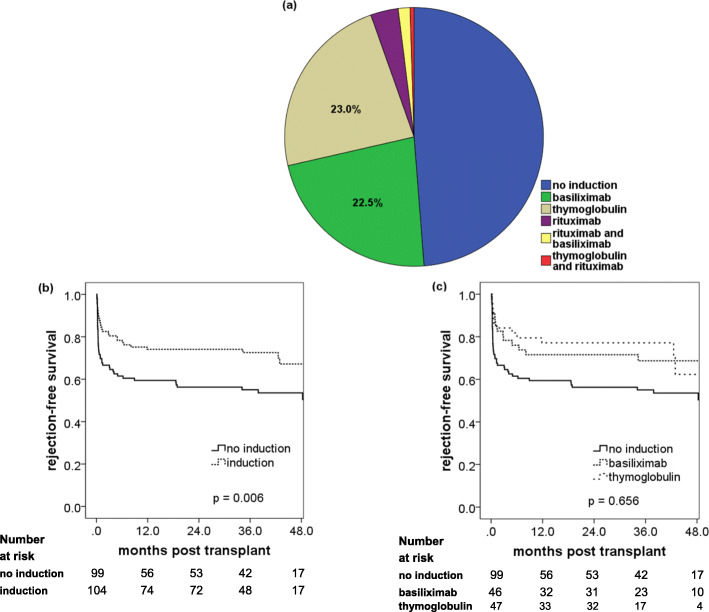
Induction therapies and rejection-free survival. **a** Distribution of the different induction therapies in the whole study group (*n* = 204 patients). **b** Comparison of acute rejection-free survival between patients with and without any type of induction therapy. **c** Comparison of acute rejection-free survival between the two most important induction therapies thymoglobulin and basiliximab (*p* = 0.656). Acute rejection rates after 12 months were 22.9% for thymoglobulin, 28.4% for basiliximab and 40.6% for the no induction group

We took a closer look at different induction therapies. Approximately half of the patients received no induction (all non-sensitized patients receiving their first transplant: immunosuppression regimen 1 in Table 1), basiliximab and thymoglobulin were roughly equally distributed (Fig. 4a). ABO incompatible patients received rituximab, combinations of different induction agents were very rarely used. Induction therapy turned out to be the most important factor in preventing acute rejection, since patients with any type of induction performed better than patients without induction (Fig. 4b, *p* = 0.006). Between the two most frequently used induction therapies (basiliximab and thymoglobulin), no difference was found in acute rejection rates (Fig. 4c, *p* = 0.656).

### Allograft function after 1 year

The median of CKD stage overall 1 year after transplantation was 2 (25. percentile = 1, 75. percentile = 3), and no difference was found between DSA positive and DSA negative patients (*p* = 0.226). Concerning proteinuria after 1 year there was also no difference between the groups.

### Comparison with historical controls at our center

For a comparison of AMR incidence in sensitized recipients with a period before introduction of thymoglobulin induction, we used our own historical data, which were collected between 2005 and 2008 and published in 2010 [7]. Whereas in the study of Riethmüller et al. DSA positive patients experienced a significantly higher incidence of AMR than DSA negative patients (Table 3, *p* < 0.001), this difference could not be seen after the introduction of thymoglobulin induction therapy in the current study (Table 3, *p* = 0.332).

**Table 3:** Incidence of acute rejection in current population and historical controls

	Historical controls [7]			This study		
	No DSA (*n* = 135)	With DSA (*n* = 20)	*P*-value	No DSA (*n* = 168)	With DSA (*n* = 35)	*P*-value
AMR, n (%)	1 (0.7)	7 (35.0)	< 0.001	13 (7.7)	4 (11.4)	0.332
TMR, n (%)	31 (22.9)	8 (40.0)	0.090	58 (34.5)	7 (20.0)	0.067

*HLA* Hyuman leukocyte antigens, *DSA* Donor-specific antibody, *AMR* Antibody-mediated rejection, *TMR* T-cell-mediated rejection

The incidence of AMR in this high risk group was reduced from 35% in the historical controls to 11.4% in the current study. Similarly, the incidence of T cell-mediated rejection (TMR) was reduced from 40 to 20% (Table 3). Thus, thymoglobulin induction had a protective effect against early acute rejection (AMR and TMR) in immunologically high risk patients with DSA.

### Infectious complications

The most common events were bacterial infections and viral infections with CMV or BK polyomavirus. Overall 33% of patients experienced bacterial infections leading to hospitalization. Furthermore, 70% suffered from viremia in the first year: 49.3% with CMV and 28.6% with BK polyomavirus. As shown in Table 4, we found that patients who received induction therapy did not experience more infections than those who had no induction. Also, the number of hospitalizations due to infections was comparable with or without induction therapy except for CMV viremia, which was lower in basiliximab-treated patients during the first year (Table 4, *p* = 0.023).

**Table 4:** Infectious complications

	Type of induction therapy^a^			
	No induction (*n* = 99)	Basiliximab (*n* = 46)	Thymoglobulin (*n* = 47)	*P*-value
Bacterial infection, n (%)	35 (36.8)	13 (28.9)	16 (36.4)	0.633
Viremia, n (%)				
Cytomegalovirus	59 (61.5)	17 (37.0)	23 (51.1)	0.023
BK Polyomavirus	25 (26.0)	15 (32.6)	17 (37.8)	0.346
Hospitalisation due to				
Infection, n (%)	37 (38.9)	14 (31.1)	18 (40.9)	0.581

^a^Combinations of different induction therapies (*n* = 4) and induction with rituximab (*n* = 7) were not included in the statistical evaluation because of the small number

## Discussion

Despite modern immunosuppression 5–10% of renal transplant patients still experience acute rejection leading to an overall reduced graft survival [1–3]. There are currently no randomized controlled trials defining the optimal immunosuppressive strategy in immunologically high-risk recipients. This study retrospectively tested the efficacy of a tailored immunosuppression approach in kidney transplant recipients with risk-stratified treatment protocols for five different patient categories in our center. The main findings are the following: (1) we observed excellent patient and allograft survival rates at 1 and 4 years post-transplant; (2) we found no differences between sensitized patients (ABO compatible versus incompatible, DSA positive versus DSA negative) regarding acute rejection rates and graft survival; (3) compared with a historical control group at our center there was a significant reduction of antibody- and T cell- mediated rejection in sensitized thymoglobulin-treated patients; (4) there was no increased rate of infections leading to hospitalization among patients receiving thymoglobulin, but an increased incidence of CMV viremia, as confirmed by a recent Cochrane database review [22, 23].

The optimal treatment strategy for patients sensitized to the HLA antigens of their donor is unclear. In theory, the best option is avoiding transplantation across DSA, a strategy which can be achieved by paired kidney donation in living donor transplantation [10] and by acceptable mismatch programs in deceased donor transplantation [24]. But even after implementing such programs a patient population remains, where no alternative option to crossing DSA exists. Whereas most centers are reluctant to transplant across a positive CDC-XM, they may accept an organ with a negative CDC-XM, but presence of DSA up to a certain MFI value. However, it is unclear what the optimal immunosuppressive strategy in this situation is. Some centers have suggested induction therapy with rituximab and/or intravenous immunoglobulins [25–27], but other reports showed a limited efficacy of this strategy in terms of prevention of AMR and graft survival [28, 29]. Alternatively, some centers have reported a reduction in acute rejection rate by the use of thymoglobulin induction therapy in this patient population [13, 14, 30–32]. For example, the Basle group showed a significant reduction of clinical and subclinical AMR and TMR in their patient groups which however was monitored by protocol biopsies [14]. In contrast to our study, they used thymoglobulin combined with high dose intravenous immunoglobulins, whereas in our study only thymoglobulin was used. The effect on the incidence of clinical acute rejection was comparable suggesting that adding intravenous immunoglobulins might not be necessary. However, we did not perform protocol biopsies and therefore cannot argue concerning subclinical rejection episodes.

ABO blood group-incompatible kidney transplantation has been implemented in many centers around the world including also 5 of the 6 kidney transplant centers in Switzerland. We previously reported the Swiss experience, which showed excellent results in patient and death-censored graft survival [10], although a higher rate of mild cellular rejection was observed with late steroid withdrawal [17]. Here, we show our results in Zurich with absolute identical outcomes in patient and allograft survival with ABO-compatible transplants in an unselected patient population.

The strengths of our study are (1) a highly standardized, single center patient follow-up of nearly 100% up to 1 year; (2) the use of a defined and uniform risk-adapted immunosuppressive concept in our center, tightly overviewed by one of us (T.F.); (3) the availability of results of pretransplant anti-HLA antibodies based on Luminex SAB analysis in 100% of patients. However, our study also has two main limitations: first, the overall rate of acute rejections was relatively high. The main reason for this was that in non-sensitized patients receiving their first transplant no induction therapy was given. This concept has subsequently been changed in our center after 2011. The second limitation is that the study was not randomized. However, the nearly 100% follow-up of our patient population, the detailed subgroup analyses, the direct comparison to historical controls at the same center in the immediately preceding time period, provide clinical data representing real life transplant medicine without any particular patient selection apart from the exclusion of pediatric patients. The third limitation consists in the fact that some subgroups were limited in size, which is only partially compensated by a very homogeneous and highly structured follow-up of these patients in a tightly supervised single-center setting.

## Conclusions

In conclusion, a tailored immunosuppression using five different immunosuppression regimens for specific patient categories was efficient and increased the graft- and rejection-free- survival in sensitized patients.

## Supplementary Information

**Additional file 1.**

## Data Availability

The data for this retrospective study were all available in the medical records of division of nephrology at the University Hospital Zurich.

## Abbreviations

AMR: Antibody-mediated rejection
BMI: Body mass index
CDC: Complement-dependent cytotoxicity
CKD: Chronic kidney disease
CMV: Cytomegalovirus
DSA: Donor-specific antibodies
GFR: Glomerular filtration rate
HLA: Human leukocyte antigen
MFI: Mean fluorescence intensity
MMF: Mycophenolate-mofetil
PCR: Polymerase chain reaction
TMR: T cell-mediated rejection
XM: Crossmatch
