# Supplementary material for: Tailored immunosuppression after kidney transplantation - a single center real-life experience
Source: BMC Nephrol. 2020 Nov 23;21:501. doi: 10.1186/s12882-020-02137-5 (PMC7686677; doi:10.1186/s12882-020-02137-5)
Supplement: Supplementary file 1 — Additional file 1. [file 12882_2020_2137_MOESM1_ESM.docx]

**Supplementary Table 1 – MFI data of donor-specific anti-HLA antibodies**

| patient | DSA 1 (MFI)  (n=17) | DSA 2 (MFI)  (n=25) | Cumulative MFI per patient |
| --- | --- | --- | --- |
| 1 |  | DQ8/663 | 663 |
| 2 |  | DQ7/645 | 645 |
| 3 |  | DR52/3231 | 3231 |
| 4 |  | DR53/884, DQ7/888 | 1772 |
| 5 |  | DR17/5196, DR52/1394, DQ2/1343 | 7933 |
| 6 |  | DR1/3771, DQ5/748 | 4519 |
| 7 |  | DR12/523, DQ7/4967 | 5490 |
| 8 |  | DR4/640, DQ7/786 | 1426 |
| 9 | A2/656 | DR13/1233, DQ6/2526 | 4415 |
| 10 | A2/644 | DQ6/2241 | 2885 |
| 11 |  | DR11/3916, DQ7/9033 | 12949 |
| 12 | A32/5257, B27/1241 |  | 6498 |
| 13 | A2/6953, B44/5132, B8/7031 |  | 19116 |
| 14 | A24/544 | DR4/500 | 1044 |
| 15 |  | DQ6/940 | 940 |
| 16 |  | DQ2/6515 | 6515 |
| 17 |  | DR4/691 | 691 |
| 18 | A68/506 |  | 506 |
| 19 | A2/7006, B57/2035, B7/3089 | DR7/1276 | 13406 |
| 20 | B18/1040 |  | 1040 |
| 21 | A2/756 |  | 756 |
| 22 |  | DR15/727 | 727 |
| 23 | A11/1115 |  | 1115 |
| 24 | A26/1581 |  | 1581 |
| 25 | A2/2612, B7/2186, B8/3374 | DQ6/6450 | 14622 |
| 26 |  | DR17/679 | 679 |
| 27 | A23/902, A25/1118 | DQ5/3438 | 5458 |
| 28 |  | DR4/703, DQ2/811 | 1514 |
| 29 |  | DQ7/856, DQ2/1614 | 2470 |
| 30 | A31/1263 | DR7/4987 | 6250 |
| 31 | B18/3216 |  | 3216 |
| 32 |  | DR11/759, DR52/1679 | 2438 |
| 33 | A3/732 |  | 732 |
| 34 |  | DR7/511, DR53/14596 | 15107 |
| 35 | B18/8340 |  | 8340 |

MFI mean-fluorescence intensity; DSA, donor-specific antibody; n, number

**Supplementary Table 2 – isoagglutinine titers in ABO-incompatible transplantations**

| patient  (n=14) | recipient  (blood group) | donor  (blood group) | IgM  (Isoagglutinin titer) | IgG  (Isoagglutinin titer) |
| --- | --- | --- | --- | --- |
| 1 | 0 | A | 1/64 | 1/64 |
| 2 | A | B | 1/32 | 1/32 |
| 3 | A | AB | 1/64 | 1/32 |
| 4 | 0 | A | 1/64 | 1/64 |
| 5 | A | B | 1/128 | 1/64 |
| 6 | 0 | A | 1/128 | 1/256 |
| 7 | 0 | A | 1/128 | 1/256 |
| 8 | 0 | A | 1/64 | 1/512 |
| 9 | 0 | A | 1/16 | 1/16 |
| 10 | 0 | A | 1/16 | 1/32 |
| 11 | 0 | B | 1/32 | 1/64 |
| 12 | 0 | A | 1/32 | 1/32 |
| 13 | 0 | A | 1/64 | 1/64 |
| 14 | A | B | 1/256 | 1/32 |

AB0i, AB0 incompatibility; n, number; BG, blood group; IgM, immunoglobulin M;

IgG, immunoglobulin G
